# Oral health care for the critically ill: a narrative review

**DOI:** 10.1186/s13054-021-03765-5

**Published:** 2021-10-01

**Authors:** Lewis Winning, Fionnuala T. Lundy, Bronagh Blackwood, Daniel F. McAuley, Ikhlas El Karim

**Affiliations:** 1 Dublin Dental University Hospital, Trinity College Dublin, Dublin, Ireland; 2 Centre for Experimental Medicine, School of Medicine, Dentistry and Biomedical Sciences, Queen’s University Belfast, The Wellcome-Wolfson Building, 97 Lisburn Road, Belfast, BT9 7AE Northern Ireland UK

**Keywords:** VAP, Oral health, Chlorhexidine, Oral bacteria, Pneumonia

## Abstract

**Background:**

The link between oral bacteria and respiratory infections is well documented. Dental plaque has the potential to be colonized by respiratory pathogens and this, together with microaspiration of oral bacteria, can lead to pneumonia particularly in the elderly and critically ill. The provision of adequate oral care is therefore essential for the maintenance of good oral health and the prevention of respiratory complications.

**Main body:**

Numerous oral care practices are utilised for intubated patients, with a clear lack of consensus on the best approach for oral care. This narrative review aims to explore the oral-lung connection and discuss in detail current oral care practices to identify shortcomings and offer suggestions for future research. The importance of adequate oral care has been recognised in guideline interventions for the prevention of pneumonia, but practices differ and controversy exists particularly regarding the use of chlorhexidine. The oral health assessment is also an important but often overlooked element of oral care that needs to be considered. Oral care plans should ideally be implemented on the basis of an individual oral health assessment. An oral health assessment prior to provision of oral care should identify patient needs and facilitate targeted oral care interventions.

**Conclusion:**

Oral health is an important consideration in the management of the critically ill. Studies have suggested benefit in the reduction of respiratory complication such as Ventilator Associated Pneumonia associated with effective oral health care practices. However, at present there is no consensus as to the best way of providing optimal oral health care in the critically ill. Further research is needed to standardise oral health assessment and care practices to enable development of evidenced based personalised oral care for the critically ill.

## Introduction

The oral cavity houses the second largest microbiota in the human body and includes bacteria, fungi, viruses, and archaea [1]. The majority of micro-organisms within the oral cavity are found within biofilms consisting of mostly commensal bacteria that are considered beneficial for the host. However, dysbiosis of the microbial biofilm can lead to dental diseases such as periodontitis and tooth decay [2]. Periodontitis is a chronic inflammatory disease affecting the supporting tissues of the teeth and is generally caused by oral anaerobic bacteria in a susceptible individual. The disease is highly prevalent, with severe forms affecting 10% of the population [3]. Tooth decay, on the other hand, is caused by acid produced by oral bacterial fermentation of dietary carbohydrates. Untreated dental caries is the 2nd most common chronic disease, with 2.4 billion individuals affected worldwide [4]. Untreated caries can ultimately lead to the death of the tooth and subsequent abscess formation in the underlying tissues.

Localised oral diseases, including periodontitis and caries-induced infections, have previously been shown to have systemic connections [5]. Oral bacteria commonly gain entrance to the circulation through ulcerated gingiva crevicular tissue that surrounds the teeth [6]. Invasion of the cariogenic Gram positive bacterium *Streptococcus mutans* into vascular endothelial cells is considered an exacerbating factor in infective endocarditis [7]. Additionally, oral bacteria including *Staphylococcus aureus, Streptococcus sanguis, Enterococcus faecalis*, and others have been implicated in the pathogenesis of infective endocarditis [8]. Poor oral hygiene in this regard, has been shown to be associated with an increased risk for infective endocarditis [9]. Gram negative oral bacteria and the local inflammatory response associated with periodontitis, can contribute to systemic inflammation and the initiation and progression of chronic inflammatory based diseases, including cardiovascular disease [10], diabetes [11] and respiratory disease [12].

This narrative review aims to provide an overview on the links between oral health and respiratory disease with particular consideration to the critically ill. We also consider the roles oral health assessment and oral care interventions have in the critically ill. A comprehensive search of the published English literature was conducted in PubMed, Medline, and Scopus until March 2021, using the following keywords: (“oral health” OR “oral disease” OR “periodontitis*” OR “caries” OR “oral health assessment” OR “oral health care” OR “oral prophylaxis”) AND (“critically ill” OR “critical care” OR “intensive care” OR “VAP”). Two of our investigators independently searched the databases (IEK and LW) and reviewed each of the retrieved articles.

### Oral health and respiratory disease

The airway, including upper and lower segments, are a continuum of the oro-nasopharynx. Secretions of the upper airways are normally heavily contaminated with microorganisms originating from the oro-nasopharynx region. The lower airways, however, maintain a more sterile-like state supported by the cough reflex, the action of tracheobronchial secretions, mucociliary transport of inhaled microorganisms, and immune defence factors (cell-mediated immunity, humoral immunity, and neutrophils). In individuals with underlying chronic health problems, aspirated oral secretions containing potential pathogens are not always cleared effectively [13]. In these cases, pathogenic changes to the normal commensal microflora of the respiratory system, and more specifically potential infections that are derived from the oral cavity, represent a mechanistic pathway for an association with oral health.

The oral microbiome is comprised of over 600 prevalent taxa at the species level, with distinct subsets predominating in various oral habitats [1]. Dental caries and periodontitis are the most common oral diseases and are major causes of tooth loss [3]. Despite different aetiologies, caries and periodontal disease represent dysbiotic states of the oral microbiome [14]. In the absence of effective oral hygiene, initial dental plaque formation on a clean tooth surface will occur within 48 h. As the biofilm matures, its composition reflects the oral environment. If the pH in the oral cavity is low, then a cariogenic microbiota may predominate (Gram-positive bacteria and *Candida albicans*), whereas if the gums are inflamed a periodontopathogenic microbiota is likely to predominate (anaerobic Gram-negative bacteria). Immunocompromised patients and individuals with low salivary flow rates will generally tend to be more susceptible to bacterial and fungal colonisation of the oral cavity. As well as leading to oral disease these pathogenic oral bacteria may be transported to the lungs where they have the potential to cause respiratory infections [15]. One cubic millimetre of dental plaque contains about 100 million bacteria [16], and may serve as a persistent reservoir for potential pathogens. Micro-aspiration of oral bacteria is common and frequently occurs during sleep. Studies have shown that typical aspirated volumes are of an amount likely to contain bacterial pathogens [17].

Amongst the associations between oral health and various respiratory diseases, the association with pneumonia has received much attention due to the strength of biological plausibility. Oral colonisation by respiratory pathogens, fostered by poor oral hygiene, has been associated with hospital-acquired pneumonia [12, 18]. Hospital-acquired pneumonia is typically caused by bacteria that are not normally residents of the oropharynx but enter this milieu from the environment. These include Gram-negative bacilli, *Pseudomonas aeruginosa*, *Staphylococcus aureus*, and enteric species (such as *Escherichia coli*, *Klebsiella pneumoniae, Serratia species, Enterobacter species*). In ventilator‐associated pneumonia (VAP), the placement of an endotracheal tube can transport oropharyngeal organisms into the lower airway [19]. The growth of a biofilm resistant to host defences and antibiotics, on the surface of the tube represents a further problem [20]. Recently, in an in vitro study, we showed that the opportunistic oral pathogen *C. albicans* enhanced bacterial numbers of the VAP pathogens; *E*. *coli*, *S*. *aureus* and MRSA in dual-species biofilms [21]. Studies have also linked community acquired pneumonia with poor oral hygiene [22, 23].

There have been several systematic reviews that have aimed to investigate the association between oral health and pneumonia. Khadka et al. [24] performed a systematic review which included studies investigating pathogenic microorganisms in oral specimens of older people with aspiration pneumonia. Based on twelve studies (four cross-sectional, five cohort and three intervention) it was found that colonisation of the oral cavity by microorganisms commonly associated with respiratory infections. Furthermore, aspiration pneumonia occurred less in people who received professional oral care compared with no such care. In a systematic review focusing specifically on the association between periodontitis and   nosocomial pneumonia, a meta-analysis was performed on 5 case–control studies that met the inclusion criteria [25]. A significant association was found between periodontitis and nosocomial pneumonia with an OR = 2.55, (95% CI 1.68–3.86). In a systematic review conducted by El-Rabbany et al. [26] focus was given to reviewing RCTs that evaluated the efficacy of prophylactic oral health procedures in reducing hospital-acquired pneumonia or ventilator-associated pneumonia. Twenty-eight trials were identified which found that good oral health care was associated with a reduction in the risk for hospital acquired and ventilator-associated pneumonia in high-risk patients.

### Oral health in critically ill intubated patients

Critically ill patients in the ICU represent a uniquely vulnerable group. Patients that are unconscious or sedated in ICUs often require mechanical ventilation with an associated risk of VAP. VAP significantly increases mortality and complications, resulting in an increased period of ventilation, longer ICU stay and associated increased costs [27]. It has been shown that oral health deteriorates following admission to ICU [28]. Dental plaque accumulates rapidly in the mouths of critically ill patients with a significant shift in plaque microbial community observed in mechanically ventilated patients, including colonisation with potential VAP pathogens [29, 30]. This confirmed previous findings that respiratory pathogens isolated from the lung are often genetically indistinguishable from strains of the same species isolated from the oral cavity in patients who receive mechanical ventilation [31]. Plaque accumulation is exacerbated in the absence of adequate oral care and by the drying of the oral cavity due to prolonged mouth opening, leading to severe inflammation of soft tissues. Pre-existing poor oral health on admission to ICU further complicates the picture and has been recognised as a specific risk factor in VAP development [32]. More recently, a case control study has demonstrated the impact of poor oral health in the form of periodontitis, and the associated higher risk of ICU admission, need for assisted ventilation and mortality during the COVID-19 pandemic [33].

### Oral health assessment

The oral health of intubated patients deteriorates with time in ICU and this is particularly problematic for those with pre-existing dental disease. Several studies have verified that teeth and other oral surfaces of patients in ICU subjects serve as reservoirs for respiratory pathogen colonization, with the pathogens causing pneumonia appearing to first colonize the dental plaque on teeth or dentures, rather than soft tissues [34]. In intubated patients with poor baseline dental health, such as periodontal disease and tooth decay, the dysbiotic plaque is likely to be mature and its removal requires special considerations. Oral health assessment prior to provision of oral care is therefore important to identify oral disease and subsequently target specific oral care needs. Oral health assessment is a descriptive health measurement needed to establish the patient’s baseline oral health status, changes in oral health during the course of care, and response to interventions [35]. An oral health assessment should include a general observation and an intra-oral examination to detect changes in the oral cavity, including, teeth, soft tissues and saliva [36]. The oral assessment should be performed frequently as part of a systematic patient assessment and should be used to identify those at increased risk of oral complications.

Despite the obvious benefits, an oral health assessment is not routinely performed for critically ill patients [37, 38], as the process is considered time-consuming and requires the training of nursing staff to identify oral disease. Furthermore, the tools that are available for oral assessment are variable, mostly not validated and are mostly developed for oral health assessment in different settings but adapted for use in ICU (Table 1). It is therefore not surprising that wide variability in oral care assessment practices exists [39]. In a recent consensus paper, the British Association of Critical Nurses (BACCN) emphasised the importance of oral assessment and identified the need for further research [36]. Oral care protocols that were based on an oral health assessment were previously found to be more cost-effective and resulted in a significant reduction of VAP [40–42]. As the provision of oral care for the critically ill and in particular those who are mechanically ventilated is complex and demanding, oral health assessment prior to provision of oral care to identify the oral disease and subsequent targeted oral care interventions could result in more clinically and cost-effective care [40, 41]**.**

**Table 1:** Oral health assessment tools commonly used in ICUs

Tool	Content	Measurement	Validation	Other
Beck Oral Assessment Score (BOAS) Beck [66]	lips, tongue and mucosa, gingiva, teeth and saliva	5 items each with a four-point scale 1–4 Max score 20	No	Developed for assessment of stomatitis post chemotherapy and adopted with modification for ICU
Bedside oral exam (BOE) Prendergast et al. [42]	Lips, tongue, saliva, mucous membranes, gingiva, teeth and odour	8 Items each with a three-point scale 1–3 Max score 24	Yes	Modified from the Oral Assessment Guide (OAG) developed for assessment of mucositis post radiation therapy and adopted with modification for ICU
Mucosal Plaque Score (MPS) Henriksen et al. [67]	Plaque Mucosa	1–4 Point scale for each item Max score 8	No	Developed to assess oral care in the elderly
The BRUSHED Assessment Model Hayes and Jones [68]	Bleeding, redness, ulceration, saliva, halitosis, external factors, and debris	Mnemonic to aid nursing staff in detecting clinical signs of impaired oral health	No	Its use in ICU is not well documented

### Oral care interventions for the critically ill

The importance of adequate oral care has been recognised in guideline interventions for the prevention of VAP [43]. Different oral practices have been adopted for intubated patients, including toothbrushing and the use of oral care solutions such as antiseptic mouthwash. However, the most effective way to achieve good oral care in the ICU is not known, and there is currently a lack of consensus [44].

Among oral care solutions, the oral antiseptic chlorhexidine digluconate was reported as the most widely used antiseptic for oral hygiene in European ICU patients [45]. Multiple systematic reviews including both randomised and non-randomised clinical trials have reported the effectiveness of chlorhexidine (CHX) in reducing VAP and mortality (Table 2). A recent Cochrane review performed a meta-analysis based on 18 RCTs and found that CHX reduced the risk of VAP compared to placebo or usual care from 24% to about 18% (RR 0.75, 95% confidence intervals (CI) 0.62–0.91, *P* = 0.004) [46]. Despite this, the use of CHX has been brought into question by the finding that a possible (non-significant) increase in mortality was reported [44, 47, 48]. It not clear, however how CHX increases the risk of mortality which has led to calls for further research to investigate its safety in critical care settings [49, 50]. CHX exhibits broad-spectrum antimicrobial activity and is considered stable, safe and effective in reducing plaque formation [51]. However, it has some disadvantages including, tooth discolouration and mucosal ulcerations when used in high concentrations, as well as emerging evidence of microbial resistance [52]. Furthermore, CHX has limited antimicrobial activities on established biofilms and therefore mechanical plaque removal, such as tooth brushing, is required prior to supplemental use of CHX [53, 54]. Future studies should be designed with these limitations in mind. Within the critical care context, the method of application of chlorhexidine is also worthy of consideration, as the use of gels may be safer than solutions, to reduce the risk of microaspiration.

**Table 2:** Summary of systemic reviews findings on the effect of chlorhexidine used in ICUs

Study	Intervention/comparisons	Outcomes	Relative effect	Number of participants	Grade
Zhao et al. [46] China	CHX (mouth rinse or gel) versus placebo/usual care	VAP	CHX reduced VAP: RR 0.67 (95% CI 0.47–0.97)	1206 (13 studies)	⨁⨁⨁◯ Moderate
		Mortality	No difference RR 1.03 (95% CI 0.80 to1.33)	944 (9 studies)	⨁⨁⨁◯ Moderate
		ICU stay	No difference 0.89 (95%CI-3.59–1.82)	627 (5 studies)	⨁⨁◯◯ Low
Silvestri et al. [69] Italy	CHX (0.12–0.2% solution or gel) versus placebo, usual care	Mortality	No difference OR: 0.69 (95% CI 0.31–1.53)^a^	1655 (5 studies)	Not reported
		Bloodstream infection	No difference OR: 0.74; 95% CI 0.37–1.50		
Hua et al. [63] China	CHX (mouth rinse or gel) versus placebo/usual care	VAP	CHX reduced VAP RR 0.75 (95% CI 0.62–0.91)	2451(18 studies)	⨁⨁⨁⨁ High
		Mortality	No difference RR 1.09 (95% CI 0.96–1.23)	2014(14 studies)	⨁⨁⨁◯ Moderate
		ICU stay	No difference 0.21 (95%CI -1.48 -1.89)	833 (6 studies)	⨁⨁⨁◯ Moderate
Villar et al. [70] Brazil	CHX (0.12–2% solution, gel or foam) versus placebo or usual care	VAP	No difference with 0.1 and 0.2% 2% CHX reduced VAP RR:0.53 (95% CI 0.31–0.91)	1640 (13 studies)	Not reported
Klompas et al. [47] USA	Interventions: CHX (0.12–2% solution, or gel) versus placebo/usualcare	VAP	CHX reduced VAP RR, 0.56 (95% CI, 0.41–0.77) in CS No significant difference for NCS RR, 0.88 (95% CI, 0.66–1.16)	1868 (3 studies)	Not reported
		Mortality	No difference: CS RR, 0.88 (95% CI, 0.25–2.14) NCS RR, 1.13, 95% CI, 0.99–1.29	1762 (13 studies)	Not reported
Price et al. [48] UK	SDD, SOD and topical oropharyngeal CHX versus usual care	Mortality	SDD reduced mortality OR 0.73 (95%CI 0.64–0.84)	7839 (15 studies)	Not reported
			SOD reduce mortality OR 0.85 (95%CI 0.74–0.97)	4276 (4 studies)	Not reported
			CHX increased mortality OR 1.25 9% CI 1.05–1.50	2618 (11 studies)	Not reported

CS, cardia surgery; NCS, non cardiac surgery; CHX, chlorhexidine; SDD, selective digestive decontamination; SOD, selective oropharyngeal decontamination; OR, odds ratio; RR, risk ratio

^a^Combined summary of interventions versus control

Although the adjunct use of chemical plaque control may be useful, effective control of dental plaque biofilm requires physical disruption with mechanical devices such as toothbrushing. Control of dental plaque and oral disease using mechanical means alone is well documented in the general population [55, 56]. In the critically ill, mechanical plaque control is widely used, but its efficacy in reducing the incidence of VAP is debatable. A systematic review of four RCT that included 828 patients showed toothbrushing did not significantly reduce the incidence of VAP (RR, 0.77; 95% CI 0.50–1.21) and mortality (RR, 0.88; 95% CI 0.70–1.10) [57]. On the other hand, Zhao et al., showed in a combined meta-analysis of five studies (910 participants), that toothbrushing reduced the incidence of VAP (RR 0.61, 95% CI 0.41–0.91, *P* = 0.01) [46]. In addition, toothbrushing compared to CHX was found to significantly reduce the duration of mechanical ventilation (MD − 1.46 days, 95% CI − 2.69 to − 0.23 days, *P* = 0.02) and ICU stay (MD − 1.89 days, 95% CI − 3.52 to − 0.27 days, *P* = 0.02), but had no effect on mortality (RR 0.86, 95% CI 0.70–1.05, *P* = 0.14). It is important to note here that the efficacy of toothbrushing in reducing plaque in these studies was reported in only one study [58] where the reduction in plaque scores was associated with a reduction in VAP.

Toothbrushing combined with antiseptics is a commonly used oral hygiene practice and showed efficacy in controlling plaque and periodontal disease [59]. In their meta-analysis Zhao et al. combined two studies (649 participants), investigating toothbrushing with chlorhexidine compared to chlorhexidine alone and no difference in the incidence of VAP (RR 0.74, 95% CI 0.50–1.09, *P* = 0.13), or mortality (RR 0.87, 95% CI 0.68–1.12, *P* = 0.28) was found [46]. Another systematic review compared CHX alone to oral hygiene protocols involving mechanical removal of biofilm (toothbrushing, scrapping) together with chlorhexidine [60]. Their meta-analysis of six studies (1276 patients) showed a reduction in the incidence of VAP in oral care protocols that combined mechanical plaque removal and CHX (risk difference: − 0.06 (95% CI − 0.11 to − 0.02; *P* = 0.007). CHX is known to be deactivated if used immediately following toothbrushing with toothpaste containing anionic surfactants [61] and it is not clear from these studies whether such considerations were taken into account.

### Other oral care interventions

Several other oral care solutions are used in ICU in addition to CHX. These include antiseptics such as povidone iodine, Listerine and triclosan as well as non-antiseptics such as saline and bicarbonate. In their systematic review, Zhao et al. compared povidone iodine rinse with a saline rinse or placebo in a meta-analysis of three studies (356 participants). They showed evidence of a reduction in VAP in the povidone iodine group (RR 0.69, 95% CI 0.50–0.95, *P* = 0.02). On the contrary, their meta-analysis of 4 studies, which compared a saline rinse with a saline-soaked swab, found that saline rinse may reduce the incidence of VAP (RR 0.47, 95% CI 0.37–0.62, *P* < 0.001) [46]. A recent systematic review investigating the effectiveness of novel herbal oral care products in the prevention of VAP reported comparable affects to CHX [62]. However, with only a limited number of studies investigating these products, further studies are required.

It is apparent from the discussion above that there is no clear consensus on the most clinically relevant and cost-effective oral care intervention. In an attempt to define the most effective oral care intervention for the prevention of VAP, Sankaran and Sonis [64] exploited the existing meta-analysis data of a Cochrane systematic review [63], and performed a network meta-analysis (NMA) to compare different oral care interventions across different studies and rank the efficacy of each in the context of all of the interventions studied. The NMA included 25 studies (4473 subjects), 16 treatments, 29 pairwise comparisons, and 15 designs. The results based on the NMA most frequent ranking probability scores (P) showed that tooth brushing (P fixed-0.94, P random-0.89), tooth brushing with povidone-iodine (P fixed-0.90, Prandom-0.88), and furacillin (P fixed-0.88, P random-0.84) were the best three interventions for preventing VAP. CHX of 0.2% concentration (P score fixed of 0.65, P score random of 0.65) ranked as the second-best intervention in the network along with Biotene (P score fixed of 0.59, P score random 0.54) and potassium permanganate (P score fixed of 0.53, P score random 0.54). The NMA demonstrated the superiority of toothbrushing or mechanical cleaning and when combined with a mouthwash, NMA showed that tooth brushing is superior to a mouthwash alone and toothbrushing with povidone iodine is superior to any other mouthwash. The results of this NMA are however based on a mix of low risk and high risk of bias studies and are not recommended for clinical treatment needs. High quality clinical trials are needed taking into account the outcome of this NMA to determine the best intervention taking into account patient-specific oral care needs. A further consideration, relates to potential barriers in the implementation of oral care protocols. An ethnographic investigation found that the complexity of performing oral care in ICU setting is underestimated and undervalued [65]. Technical barriers included oral crowding with tubes and aversive responses by patients such as biting. Contextual impediments to oral care included time constraints, lack of training, and limited opportunities for interprofessional collaboration.

## Conclusion

The contribution of poor oral hygiene and oral bacteria to the development of pneumonia is well established. Within the context of critical care, however, controversy exists as to the best practice to achieve optimal oral health care and whether this is reflected in better overall outcomes for ICU patients. Further research is needed to standardise oral care practices and personalise individuals’ oral health needs within the ICU.

## Data Availability

The datasets used and/or analysed during the current study are available from the corresponding author on reasonable request.

## Abbreviations

BACCN: British Association of Critical Nurses
BOAS: Beck Oral Assessment Score
BOE: Bedside oral exam
CHX: Chlorhexidine
CS: Cardiac surgery
ICUs: Intensive care units
MPS: Mucosal Plaque Score
NCS: Non cardiac surgery
NMA: Network meta-analysis
OAG: Oral Assessment Guide
OR: Odds ratio
RCTs: Randomised control trials
RR: Risk ratio
VAP: Ventilator associated pneumonia

## References

[1] Dewhirst FE, Chen T, Izard J, Paster BJ, Tanner ACR, Yu WH, Lakshmanan A, Wade WG (2010). The human oral microbiome. J Bacteriol.

[2] Marsh PD, Zaura E (2017). Dental biofilm: ecological interactions in health and disease. J Clin Periodontol.

[3] Kassebaum NJ, Bernabe E, Dahiya M, Bhandari B, Murray CJ, Marcenes W (2014). Global burden of severe periodontitis in 1990–2010: a systematic review and meta-regression. J Dent Res.

[4] Frencken JE, Sharma P, Stenhouse L, Green D, Laverty D, Dietrich T (2017). Global epidemiology of dental caries and severe periodontitis: a comprehensive review. J Clin Periodontol.

[5] Han YW, Wang X (2013). Mobile microbiome: oral bacteria in extra-oral infections and inflammation. J Dent Res.

[6] Lockhart PB, Brennan MT, Sasser HC, Fox PC, Paster BJ, Bahrani-Mougeot FK (2008). Bacteremia associated with toothbrushing and dental extraction. Circulation.

[7] Abranches J, Zeng L, Bélanger M, Rodrigues PH, Simpson-Haidaris PJ, Akin D, Dunn WA, Progulske-Fox A, Burne RA (2009). Invasion of human coronary artery endothelial cells by *Streptococcus mutans* OMZ175. Oral Microbiol Immunol.

[8] Del Giudice C, Vaia E, Liccardo D, Marzano F, Valletta A, Spagnuolo G, Ferrara N, Rengo C, Cannavo A, Rengo G (2021). Infective endocarditis: a focus on oral microbiota. Microorganisms.

[9] Lockhart PB, Brennan MT, Thornhill M, Michalowicz BS, Noll J, Bahrani-Mougeot FK, Sasser HC (2009). Poor oral hygiene as a risk factor for infective endocarditis–related bacteremia. J Am Dent Assoc.

[10] Sanz M, Marco Del Castillo A, Jepsen S, Gonzalez-Juanatey JR, D'Aiuto F, Bouchard P, Chapple I, Dietrich T, Gotsman I, Graziani F (2020). Periodontitis and cardiovascular diseases: consensus report. J Clin Periodontol.

[11] Sanz M, Ceriello A, Buysschaert M, Chapple I, Demmer RT, Graziani F, Herrera D, Jepsen S, Lione L, Madianos P (2018). Scientific evidence on the links between periodontal diseases and diabetes: Consensus report and guidelines of the joint workshop on periodontal diseases and diabetes by the International Diabetes Federation and the European Federation of Periodontology. J Clin Periodontol.

[12] Linden GJ, Lyons A, Scannapieco FA (2013). Periodontal systemic associations: review of the evidence. J Clin Periodontol.

[13] Azarpazhooh A, Leake JL (2006). Systematic review of the association between respiratory diseases and oral health. J Periodontol.

[14] Mira A, Simon-Soro A, Curtis MA (2017). Role of microbial communities in the pathogenesis of periodontal diseases and caries. J Clin Periodontol.

[15] Manger D, Walshaw M, Fitzgerald R, Doughty J, Wanyonyi KL, White S, Gallagher JE (2017). Evidence summary: the relationship between oral health and pulmonary disease. Br Dent J.

[16] MThoden van Velzenanger SK, Abraham-Inpijn L, Moorer WR (1984). Plaque and systemic disease: a reappraisal of the focal infection concept. J Clin Periodontol.

[17] Gleeson K, Eggli DF, Maxwell SL (1997). Quantitative aspiration during sleep in normal subjects. Chest.

[18] Scannapieco FA, Bush RB, Paju S (2003). Associations between periodontal disease and risk for nosocomial bacterial pneumonia and chronic obstructive pulmonary disease. A systematic review. Ann Periodontol.

[19] Safdar N, Crnich CJ, Maki DG (2005). The pathogenesis of ventilator-associated pneumonia: its relevance to developing effective strategies for prevention. Respir Care.

[20] Feldman C, Kassel M, Cantrell J, Kaka S, Morar R, Goolam Mahomed A, Philips JI (1999). The presence and sequence of endotracheal tube colonization in patients undergoing mechanical ventilation. Eur Respir J.

[21] Luo Y, McAuley DF, Fulton CR, Sá Pessoa J, McMullan R, Lundy FT (2021). Targeting *Candida albicans* in dual-species biofilms with antifungal treatment reduces *Staphylococcus aureus* and MRSA in vitro. PLoS ONE.

[22] van der Maarel-Wierink CD, Vanobbergen JNO, Bronkhorst EM, Schols JMGA, de Baat C (2013). Oral health care and aspiration pneumonia in frail older people: a systematic literature review. Gerodontology.

[23] Kaneoka A, Pisegna JM, Miloro KV, Lo M, Saito H, Riquelme LF, LaValley MP, Langmore SE (2015). Prevention of healthcare-associated pneumonia with oral care in individuals without mechanical ventilation: a systematic review and meta-analysis of randomized controlled trials. Infect Control Hosp Epidemiol.

[24] Khadka S, Khan S, King A, Goldberg LR, Crocombe L, Bettiol S (2021). Poor oral hygiene, oral microorganisms and aspiration pneumonia risk in older people in residential aged care: a systematic review. Age Ageing.

[25] Jeronimo LS, Abreu LG, Cunha FA, Lima RPE (2020). Association between periodontitis and nosocomial pneumonia: a systematic review and meta-analysis of observational studies. Oral Health Prev Dent.

[26] El-Rabbany M, Zaghlol N, Bhandari M, Azarpazhooh A (2015). Prophylactic oral health procedures to prevent hospital-acquired and ventilator-associated pneumonia: a systematic review. Int J Nurs Stud.

[27] Zimlichman E, Henderson D, Tamir O, Franz C, Song P, Yamin CK, Keohane C, Denham CR, Bates DW (2013). Health care-associated infections: a meta-analysis of costs and financial impact on the US health care system. JAMA Intern Med.

[28] Terezakis E, Needleman I, Kumar N, Moles D, Agudo E (2011). The impact of hospitalization on oral health: a systematic review. J Clin Periodontol.

[29] Sands KM, Twigg JA, Lewis MAO, Wise MP, Marchesi JR, Smith A, Wilson MJ, Williams DW (2016). Microbial profiling of dental plaque from mechanically ventilated patients. J Med Microbiol.

[30] Sands KM, Wilson MJ, Lewis MAO, Wise MP, Palmer N, Hayes AJ, Barnes RA, Williams DW (2017). Respiratory pathogen colonization of dental plaque, the lower airways, and endotracheal tube biofilms during mechanical ventilation. J Crit Care.

[31] Heo SM, Haase EM, Lesse AJ, Gill SR, Scannapieco FA (2008). Genetic relationships between respiratory pathogens isolated from dental plaque and bronchoalveolar lavage fluid from patients in the intensive care unit undergoing mechanical ventilation. Clin Infect Dis.

[32] Takahama A, de Sousa VI, Tanaka EE, Ono E, Ito FAN, Costa PP, Pedriali MBBP, de Lima HG, Fornazieri MA, Correia LS (2021). Analysis of oral risk factors for ventilator-associated pneumonia in critically ill patients. Clin Oral Invest.

[33] Marouf N, Cai W, Said KN, Daas H, Diab H, Chinta VR, Hssain AA, Nicolau B, Sanz M, Tamimi F (2021). Association between periodontitis and severity of COVID-19 infection: a case–control study. J Clin Periodontol.

[34] Terpenning M, Bretz W, Lopatin D, Langmore S, Dominguez B, Loesche W (1993). Bacterial colonization of saliva and plaque in the elderly. Clin Infect Dis.

[35] Abidia RF (2007). Oral care in the intensive care unit: a review. J Contemp Dent Pract.

[36] Collins T, Plowright C, Gibson V, Stayt L, Clarke S, Caisley J, Watkins CH, Hodges E, Leaver G, Leyland S (2021). British association of critical care nurses: evidence-based consensus paper for oral care within adult critical care units. Nurs Crit Care.

[37] Grap MJ, Munro CL (2004). Preventing ventilator-associated pneumonia: evidence-based care. Crit Care Nurs Clin North Am.

[38] Berry AM, Davidson PM (2006). Beyond comfort: oral hygiene as a critical nursing activity in the intensive care unit. Intensive Crit Care Nurs.

[39] Labeau S, Vandijck D, Rello J, Adam S, Rosa A, Wenisch C, Bäckman C, Agbaht K, Csomos A, Seha M (2008). Evidence-based guidelines for the prevention of ventilator-associated pneumonia: results of a knowledge test among European intensive care nurses. J Hosp Infect.

[40] Ames NJ, Sulima P, Yates JM, McCullagh L, Gollins SL, Soeken K, Wallen GR (2011). Effects of systematic oral care in critically ill patients: a multicenter study. Am J Crit Care.

[41] Labeau SO, Conoscenti E, Blot SI (2021). Less daily oral hygiene is more in the ICU: not sure. Intensive Care Med.

[42] Prendergast V, Kleiman C, King M (2013). The bedside oral exam and the barrow oral care protocol: translating evidence-based oral care into practice. Intensive Crit Care Nurs.

[43] Hellyer TP, Ewan V, Wilson P, Simpson AJ (2016). The Intensive Care Society recommended bundle of interventions for the prevention of ventilator-associated pneumonia. J Intensive Care Soc.

[44] Torres A, Niederman MS, Chastre J, Ewig S, Fernandez-Vandellos P, Hanberger H, Kollef M, Li Bassi G, Luna CM, Martin-Loeches I (2017). International ERS/ESICM/ESCMID/ALAT guidelines for the management of hospital-acquired pneumonia and ventilator-associated pneumonia. Eur Respir J.

[45] Rello J, Koulenti D, Blot S, Sierra R, Diaz E, De Waele JJ, Macor A, Agbaht K, Rodriguez A (2007). Oral care practices in intensive care units: a survey of 59 European ICUs. Intensive Care Med.

[46] Zhao T, Wu X, Zhang Q, Li C, Worthington HV, Hua F (2020). Oral hygiene care for critically ill patients to prevent ventilator-associated pneumonia. Cochrane Database Syst Rev.

[47] Klompas M, Speck K, Howell MD, Greene LR, Berenholtz SM (2014). Reappraisal of routine oral care with chlorhexidine gluconate for patients receiving mechanical ventilation: systematic review and meta-analysis. JAMA Intern Med.

[48] Price R, MacLennan G, Glen J (2014). Selective digestive or oropharyngeal decontamination and topical oropharyngeal chlorhexidine for prevention of death in general intensive care: systematic review and network meta-analysis. BMJ Br Med J.

[49] Cuthbertson BH, Dale CM (2021). Less daily oral hygiene is more in the ICU: yes. Intensive Care Med.

[50] Wittekamp BH, Plantinga NL (2021). Less daily oral hygiene is more in the ICU: no. Intensive Care Med.

[51] Bonez PC, dos Santos Alves CF, Dalmolin TV, Agertt VA, Mizdal CR (2013). Chlorhexidine activity against bacterial biofilms. Am J Infect Control.

[52] Saleem HG, Seers CA, Sabri AN, Reynolds EC (2016). Dental plaque bacteria with reduced susceptibility to chlorhexidine are multidrug resistant. BMC Microbiol.

[53] ten Cate JM (2006). Biofilms, a new approach to the microbiology of dental plaque. Odontology.

[54] deLacerdaVidal CF, Vidal AKdL, Monteiro JGDM, Cavalcanti A, Henriques APDC, Oliveira M, Godoy M, Coutinho M, Sobral PD, Vilela CÂ (2017). Impact of oral hygiene involving toothbrushing versus chlorhexidine in the prevention of ventilator-associated pneumonia: a randomized study. BMC Infect Dis.

[55] van der Weijden F, Slot DE (2011). Oral hygiene in the prevention of periodontal diseases: the evidence. Periodontol 2000.

[56] Slot DE, Valkenburg C, Van der Weijden GAF (2020). Mechanical plaque removal of periodontal maintenance patients: a systematic review and network meta-analysis. J Clin Periodontol.

[57] Gu WJ, Gong YZ, Pan L, Ni YX, Liu JC (2012). Impact of oral care with versus without toothbrushing on the prevention of ventilator-associated pneumonia: a systematic review and meta-analysis of randomized controlled trials. Crit Care.

[58] Yao LY, Chang CK, Maa SH, Wang C, Chen CC (2011). Brushing teeth with purified water to reduce ventilator-associated pneumonia. J Nurs Res.

[59] Figuero E, Roldán S, Serrano J, Escribano M, Martín C, Preshaw PM (2020). Efficacy of adjunctive therapies in patients with gingival inflammation: a systematic review and meta-analysis. J Clin Periodontol.

[60] Pinto A, Silva BMD, Santiago-Junior JF, Sales-Peres SHC (2021). Efficiency of different protocols for oral hygiene combined with the use of chlorhexidine in the prevention of ventilator-associated pneumonia. J Bras Pneumol.

[61] Balagopal S, Arjunkumar R (2013). Chlorhexidine: the gold standard antiplaque agent. J Pharm Sci Res.

[62] Mojtahedzadeh M (2021). Systematic review: effectiveness of herbal oral care products on ventilator-associated pneumonia. Phytother Res.

[63] Hua F, Xie H, Worthington HV, Furness S, Zhang Q, Li C: Oral hygiene care for critically ill patients to prevent ventilator‐associated pneumonia. Cochrane Database Syst Rev 2016(10). 10.1002/14651858.CD008367.pub3 PMC6460950 27778318

[64] Sankaran SP, Sonis S (2021). Network meta-analysis from a pairwise meta-analysis design: to assess the comparative effectiveness of oral care interventions in preventing ventilator-associated pneumonia in critically ill patients. Clin Oral Invest.

[65] Dale CM, Angus JE, Sinuff T, Rose L (2016). Ethnographic investigation of oral care in the intensive care unit. Am J Crit Care.

[66] Beck S (1979). Impact of a systematic oral care protocol on stomatitis after chemotherapy. Cancer Nurs.

[67] Henriksen BM, Ambjørnsen E, Axéll TE (1999). Evaluation of a mucosal-plaque index (MPS) designed to assess oral care in groups of elderly. Spec Care Dentist.

[68] Hayes J, Jones C (1995). A collaborative approach to oral care during critical illness. Dent Health.

[69] Silvestri L, Weir WI, Gregori D, Taylor N, Zandstra DF, van Saene JJM, van Saene HKF (2017). Impact of oral chlorhexidine on bloodstream infection in critically Ill patients: systematic review and meta-analysis of randomized controlled trials. J Cardiothorac Vasc Anesth.

[70] Villar CC, Pannuti CM, Nery DM, Morillo CM, Carmona MJ, Romito GA (2016). Effectiveness of intraoral chlorhexidine protocols in the prevention of ventilator-associated pneumonia: meta-analysis and systematic review. Respir Care.

